# Well-tolerated Spirulina extract inhibits influenza virus replication and reduces virus-induced mortality

**DOI:** 10.1038/srep24253

**Published:** 2016-04-12

**Authors:** Yi-Hsiang Chen, Gi-Kung Chang, Shu-Ming Kuo, Sheng-Yu Huang, I-Chen Hu, Yu-Lun Lo, Shin-Ru Shih

**Affiliations:** 1 Research Centre for Emerging Viral Infections,College of Medicine, Chang Gung University, Taoyuan, Taiwan, ROC; 2 Department of Medical Biotechnology and Laboratory Science, College of Medicine, Chang Gung University, Taoyuan, Taiwan, ROC; 3 Graduate Institute of Biomedical Sciences, College of Medicine, Chang Gung University, Taoyuan, Taiwan, ROC; 4 Clinical Virology Lab, Department of Clinical Pathology, Chang Gung Memorial Hospital, Taoyuan, Taiwan, ROC; 5 Institute of Bioinformatics and Structural Biology, National Tsing-Hua University, Hsinchu, Taiwan, ROC

## Abstract

Influenza is one of the most common human respiratory diseases, and represents a serious public health concern. However, the high mutability of influenza viruses has hampered vaccine development, and resistant strains to existing anti-viral drugs have also emerged. Novel anti-influenza therapies are urgently needed, and in this study, we describe the anti-viral properties of a Spirulina (*Arthrospira platensis*) cold water extract. Anti-viral effects have previously been reported for extracts and specific substances derived from Spirulina, and here we show that this Spirulina cold water extract has low cellular toxicity, and is well-tolerated in animal models at one dose as high as 5,000 mg/kg, or 3,000 mg/kg/day for 14 successive days. Anti-flu efficacy studies revealed that the Spirulina extract inhibited viral plaque formation in a broad range of influenza viruses, including oseltamivir-resistant strains. Spirulina extract was found to act at an early stage of infection to reduce virus yields in cells and improve survival in influenza-infected mice, with inhibition of influenza hemagglutination identified as one of the mechanisms involved. Together, these results suggest that the cold water extract of Spirulina might serve as a safe and effective therapeutic agent to manage influenza outbreaks, and further clinical investigation may be warranted.

Influenza is one of the most common human respiratory illnesses^1^, and is known for its high morbidity and mortality, particularly in the very young, the elderly, and the chronically ill. Influenza viruses cause seasonal outbreaks every year, as well as occasional pandemics that can spread rapidly and infect up to 50% of affected populations^1^. The World Health Organization (WHO) has estimated that, worldwide, 3–5 million cases of severe illness and 250,000–500,000 deaths each year can be attributed to seasonal influenza^2^, while pandemics may exact an even greater toll^3^,^4^. Aside from mortality, influenza also inflicts a heavy economic and medical burden on society, and represents a serious public health problem that needs to be effectively addressed.

Of the influenza viruses, influenza A virus is the only type known to cause pandemics^5^. Influenza A virus strains are typically classified according to the antigenicity of their hemagglutinin (HA) and neuraminidase (NA) surface glycoproteins. HA, of which there are 18 known subtypes (H1–H18)^1^,^5^, is essential for infection, as it regulates viral binding to host cell receptors, and facilitates the fusion of viral and endosomal membranes to allow entry of the viral genome into host cells^6^. NA, of which 11 subtypes (N1–N11) have been identified^5^, is important for viral replication, as it cleaves the sialic acid groups binding newly produced viral particles to the surface of host cells, thereby releasing the viruses to infect other hosts^5^. Influenza virulence is likely driven by both high viral loads and an overly aggressive host immune response, which combine to induce extensive inflammation and apoptosis^7^,^8^. Anti-viral drugs such as M2 ion channel inhibitors (e.g. amantadine, rimantadine) or NA inhibitors (e.g. oseltamivir, zanamivir) are currently available for the prevention or treatment of influenza, but the emergence of resistant viral strains may eventually limit their efficacy^6^. Vaccination is the most effective strategy for influenza prevention, but timely vaccine development remains challenging, due to the difficulty of predicting the exact viral strain that could emerge to cause the next seasonal outbreak or pandemic. Therefore, novel anti-influenza therapies that have efficacy against a broad range of influenza virus strains are urgently needed, to improve patient outcomes and successfully manage outbreaks.

Spirulina (*Arthrospira platensis*), is a free-floating filamentous cyanobacterium that has a long history of recorded use. There is evidence that the Mayan and Aztec civilizations widely consumed dried Spirulina cakes in pre-Columbian times, and for centuries, communities in Central Africa have harvested Spirulina from the waters of Lake Chad for use as food^9^,^10^. Spirulina has received considerable attention for its 60–70% protein content and high concentrations of phenolic acids, tocopherols, essential fatty acids, and B vitamins^10^,^11^. Extracts of *Spirulina* have been reported to have multiple therapeutic effects, including cholesterol reduction, immunomodulation, antioxidant, anti-cancer, and anti-viral effects^11^.

In this study, we conducted cellular assays and animal studies to assess the effects of an *A. platensis* cold water extract (Spirulina extract) upon the viability and pathogenicity of several influenza A viral strains. Our results showed that this Spirulina extract inhibited viral plaque formation and reduced viral replication in cell cultures, and was shown to be safe and well-tolerated at high doses in cellular and animal toxicity studies. Furthermore, influenza-infected mice given Spirulina extract had higher survival rates compared to vehicle-treated controls. We further sought to identify the underlying mechanisms involved, and found that the Spirulina extract disrupted the hemagglutination of viral particles to erythrocytes, thus inhibiting the infection process. Together, these results suggest that the cold water extract of Spirulina may warrant further clinical investigation as a potential therapy for the management of influenza.

## Results

### Spirulina extract inhibits plaque formation in a broad range of influenza strains

**Figure 1:**
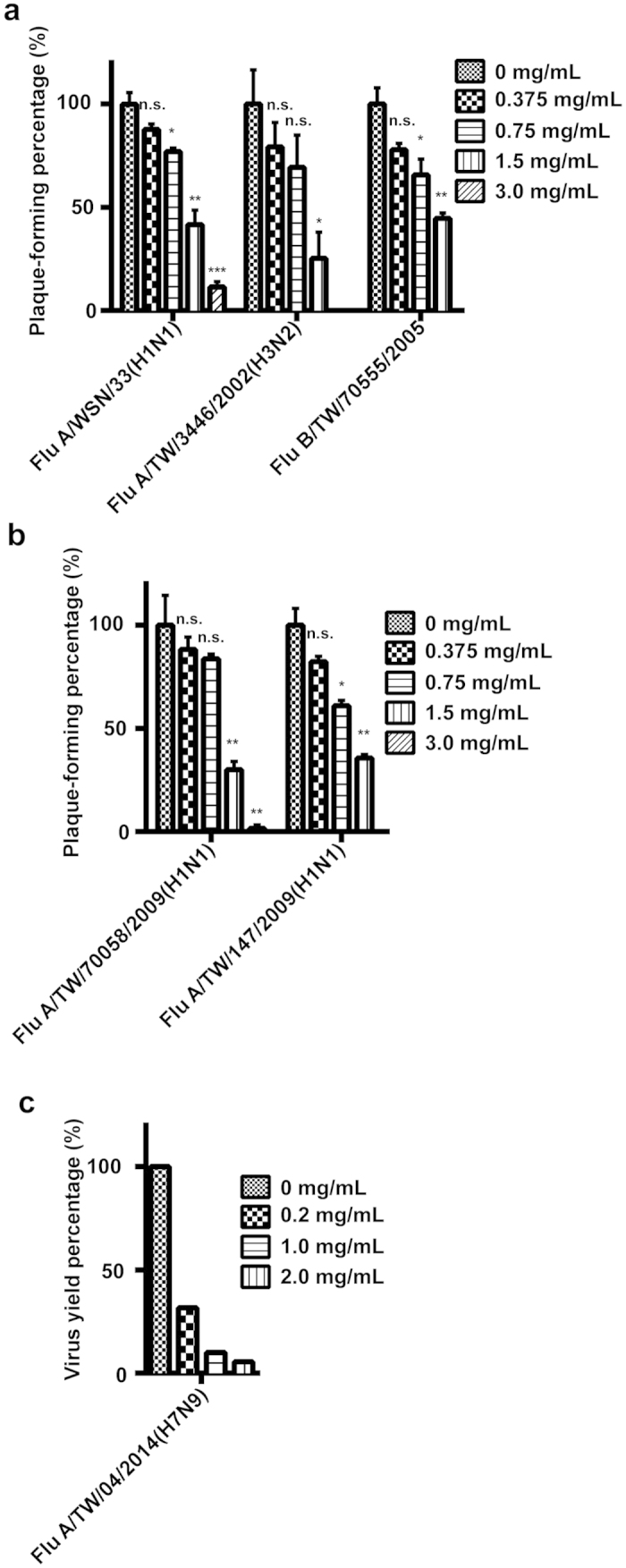
The cold water extract of Spirulina (*Arthrospira platensis*) inhibits viral replication and plaque formation in a broad range of influenza strains *in vitro*. (**a**) The influenza virus strains A/WSN/33(H1N1), A/TW/3446/02(H3N2), and B/TW/70555/05, and (**b**) the oseltamivir-resistant strains A/TW/70058/09(H1N1) and A/TW/147/09(H1N1), propagated on MDCK cells, all exhibited dose-dependent reductions in virus plaque formation after treatment with 0.375, 0.75, 1.5, and 3.0 mg/ml of Spirulina extract. Data represent the mean ± SD for three independent experiments. Statistical significance was determined by two-tailed unpaired t-test, and used as the basis to label results as ns, not significant; *P ≤ 0.05; **P ≤ 0.01; or ***P ≤ 0.001. (**c**) The TCID_50_ assay showed that Spirulina extract reduced virus production of the influenza A/TW/04/2014 (H7N9) virus.

We initially assessed the antiviral properties of Spirulina extract via plaque reduction assay. Monolayer MDCK cells cultured in 6-well plates were respectively infected with ~50 plaque-forming units (P.F.U.)/well of the influenza A/WSN/33(H1N1), A/TW/3446/02(H3N2), or B/TW/70555/05 virus strains (Fig. 1a), as well as the oseltamivir-resistant influenza A/TW/70058/2009(H1N1) or A/TW/147/2009(H1N1) viruses (Fig. 1b). The cells were treated with 0.375, 0.75, 1.5, or 3.0 mg/mL of Spirulina extract which were positioned at suitable intervals around the rough EC_50_ of 0.955 mg/ml as determined from the previous neutralization test against influenza A/WSN/33(H1N1), the same time as virus was added, and then incubated for 48 hours, after which cells were fixed and stained, and viral plaques were quantified. Compared to untreated controls, the addition of 0.375, 0.75, 1.5, or 3 mg/mL of Spirulina extract respectively led to 12.12%, 22.90%, 58.73%, or 89.00% viral plaque inhibition for the A/WSN/33(H1N1) strain, with comparable dose-dependent inhibition rates observed for other viral strains tested, including the oseltamivir-resistant strains (Fig. 1a,b). Importantly, the 3 mg/mL of Spirulina extract completely inhibited viral plaque formation in the A/TW/3446/02(H3N2), B/TW/70555/05, and oseltamivir-resistant A/TW/147/09(H1N1) strains (Fig. 1a,b). Using the results of the plaque reduction assay and the nonlinear regression function on GraphPad Prism software, we determined Spirulina extract EC_50_ values to be 1.17 ± 0.09 mg/mL for strain A/WSN/33(H1N1), 0.98 ± 0.24 mg/mL for strain A/TW/3446/02(H3N2), 1.33 ± 0.15 mg/mL for strain B/TW/70555/05, 1.17 ± 0.09 mg/mL for strain A/TW/70058/09(H1N1), and 0.98 ± 0.10 mg/mL for strain A/TW/147/09(H1N1) (Table 1). We further evaluated the cytotoxicity of the Spirulina extract upon MDCK cells with the MTT assay, and found the CC_50_ to be 23.8 mg/mL; the selectivity index (SI), a ratio of CC_50_ to EC_50_, was therefore calculated to be 20.34 for strain A/WSN/33(H1N1), 24.28 for strain A/TW/3446/02(H3N2), 17.89 for strain B/TW/70555/05, 20.34 for strain A/TW/70058/09(H1N1), and 24.28 for strain A/TW/147/09(H1N1) (Table 1). In addition, virus yield reduction determined by tissue culture infective dose (TCID_50_) results indicated that 0.2, 1.0, or 2.0 mg/mL of Spirulina extract co-incubated with the influenza A/TW/04/2014(H7N9) virus for 60 mins, then added to the MDCK cell together for 72 hours were respectively able to inhibit 68.36%, 90%, or 94% of virus yields (Fig. 1c). An influenza neutralization test further showed that the EC_50_ of Spirulina extract for the 2009 influenza pandemic strain A/TW/126/2009(H1N1) was 0.585 ± 0.02 mg/mL (Table 1.). These results demonstrate that Spirulina extract is capable of inhibiting viral replication and plaque formation in a broad range of influenza virus strains, including strains that display resistance to oseltamivir.

### Spirulina extract is safe and well-tolerated at high doses

**Figure 2:**
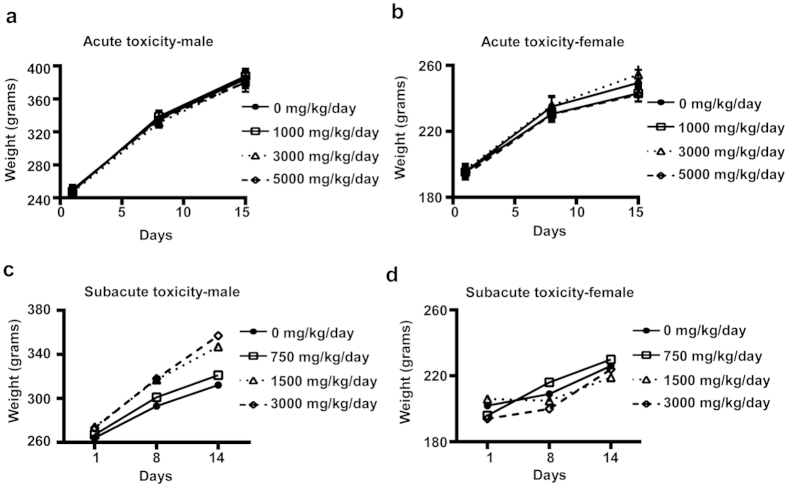
Short-term acute and subacute oral toxicity for the Spirulina extract. (**a**) 14-day acute oral toxicity study was conducted in Sprague-Dawley rats given 0, 1,000, 3,000, or 5,000 mg/kg of Spirulina extract. Body weight changes were tracked for (**a**) male and (**b**) female rats during the study period, and no significant changes were observed. (**a**) 14-day subacute study, in which Sprague-Dawley rats were subjected to repeated dosing with 750, 1,500, or 3,000 mg/kg/day of Spirulina extract for 14 consecutive days, was also conducted. Body weight changes for (**c**) male and (**d**) female rats were measured on Day 1, Day 8, and Day 14. All data are presented as mean ± SD (N = 10).

Since the Spirulina extract was shown to have broad-spectrum anti-flu activities in cellular assays, we further sought to evaluate the bio-safety of Spirulina extract *in vivo*. We performed a 14-day acute oral toxicity study in Sprague-Dawley rats, using doses of 0, 1,000, 3,000, or 5,000 mg/kg of Spirulina extract. Clinical signs, body weight, and mortality were monitored for 15 days, and body weight changes were separately tracked for male (Fig. 2a) and female (Fig. 2b) rats. Results showed no significant differences in mortality, body weight, or overall body weight change for treated and untreated controls. No lesions were found in any rats during necropsies conducted at the end of the study. From these results, we determined that the 14-day median lethal dose (LD_50_) of Spirulina extract is likely to be higher than 5,000 mg/kg. We then performed a 14-day repeated dose (subacute) oral toxicity study in Sprague-Dawley rats, which were consecutively given 750, 1,500, or 3,000 mg/kg/day of Spirulina extract for 14 days. Body weight was monitored on Day 1, Day 8, and Day 14, and changes were separately tracked for male (Fig. 2c) and female (Fig. 2d) rats. No deaths were observed in any rats during the course of the study; moreover, no significant differences in body weight were seen between treated and untreated groups. Ophthalmologic examinations, urinalysis, haematology assays (Supplementary Table S1), blood coagulation assays, and serum chemistry assays (Supplementary Table S2) were also conducted, and no significant disparities were observed for the treated groups, except for the fact that aspartate transaminase (AST) and alanine transaminase (ALT) levels decreased in groups treated with 3,000 mg/kg/day of Spirulina extract (Supplementary Table S2). According to these results, we concluded that the no observed adverse effect level (NOAEL) for Spirulina extract can be as high as 3,000 mg/kg/day. These findings show that Spirulina extract is safe and well-tolerated, even when very high doses are administered within a short period of time. The mutagenecity of the Spirulina extract was also evaluated. The Ames test, using *Salmonella typhimurium*, was conducted to assess genotoxicity for up to 5,000 μg/mL of Spirulina extract. No reverse mutation was observed at the high doses tested. A chromosome aberration assay was also conducted in Chinese hamster ovary (CHO) cells, using up to 5,000 μg/mL of Spirulina extract, and no chromosome aberrations were detected for the doses tested.

### Spirulina extract disrupts viral replication most effectively during early infection

**Figure 3:**
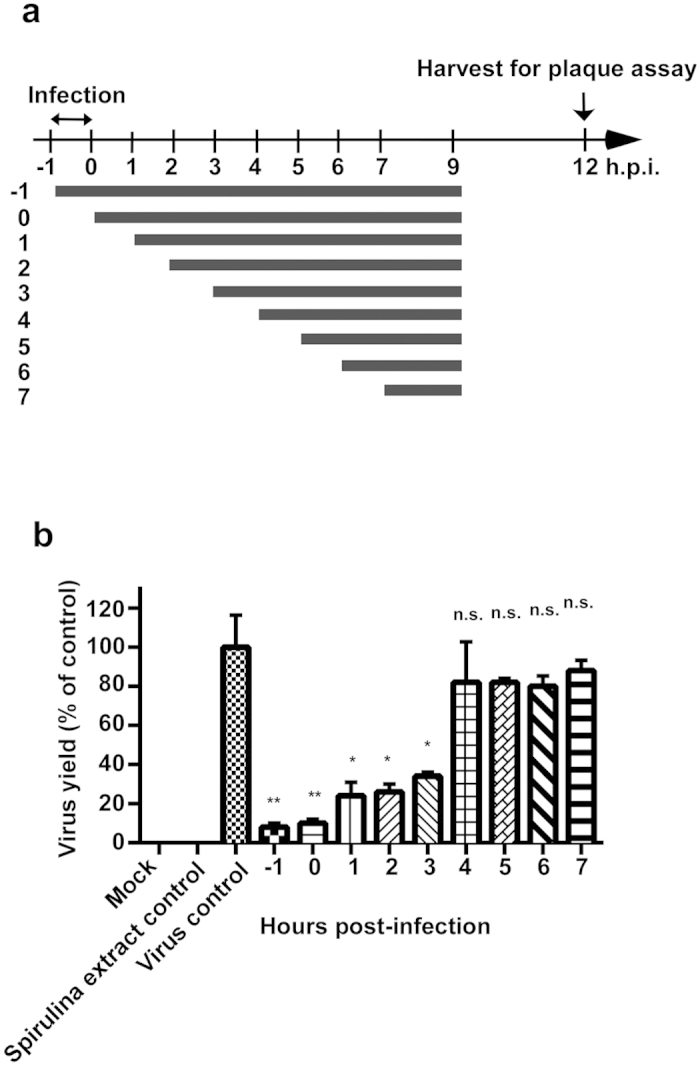
Spirulina extract acts to disrupt viral replication at an early stage of infection. (**a**) A time of addition assay was conducted, in which 2.5 mg/mL of Spirulina extract was added at the indicated timepoints to MDCK cells infected with the A/WSN/33(H1N1) influenza strain. E_0_ medium was overlaid on cultures at 9 hours post-infection, and supernatants were harvested at 12 hours post-infection. (**b**) Virus yields were determined by the plaque assay, and reported as a percentage of untreated controls. Data represent the mean ± SD for two independent experiments. Statistical analysis was performed with two-tailed unpaired t-test, and the statistical significance was determined to be ^*^P ≤ 0.05, ^**^P ≤ 0.01, or ^***^P ≤ 0.001 for each treated group versus the virus control.

As the Spirulina extract demonstrated a broad spectrum of anti-influenza activity *in vitro*, and was found to have low toxicity in cellular models, in addition to being highly tolerated in animal models, we further performed a time of addition assay to assess the effect of Spirulina extract on influenza virus replication at different stages of influenza infection, to investigate the possible anti-flu mechanisms of Spirulina extract. MDCK cells were infected with strain A/WSN/33(H1N1) at a multiplicity of infection (MOI) of 2, and then treated with 2.5 mg/mL of Spirulina extract at different time points post-infection (Fig. 3a). At 9 hours post-infection, the culture medium was replaced with E_0_ overlay medium, and supernatants were subsequently collected at 12 hours post-infection. Virus titers were assessed using plaque assays as previously described^12^, and results revealed that Spirulina extract inhibited virus yields more effectively when applied at the earlier stages of virus infection (Fig. 3b). Compared to untreated controls, 90% of virus yields were inhibited when the Spirulina extract was added at one hour prior to infection (−1) or immediately after infection (0). In contrast, 76%, 74%, and 66% inhibition of virus yields were respectively observed when Spirulina extract was added at 1, 2, and 3 hours post-infection, and when Spirulina extract was added at 4, 5, 6, and 7 hours after infection, only 18%, 18%, 20%, and 12% inhibition of virus yields were respectively observed (Fig. 3b). These results suggest that the Spirulina extract acts by disrupting the influenza infection process or by preventing viral replication in host cells.

### Spirulina extract targets hemagglutinin to inhibit influenza virus

**Figure 4:**
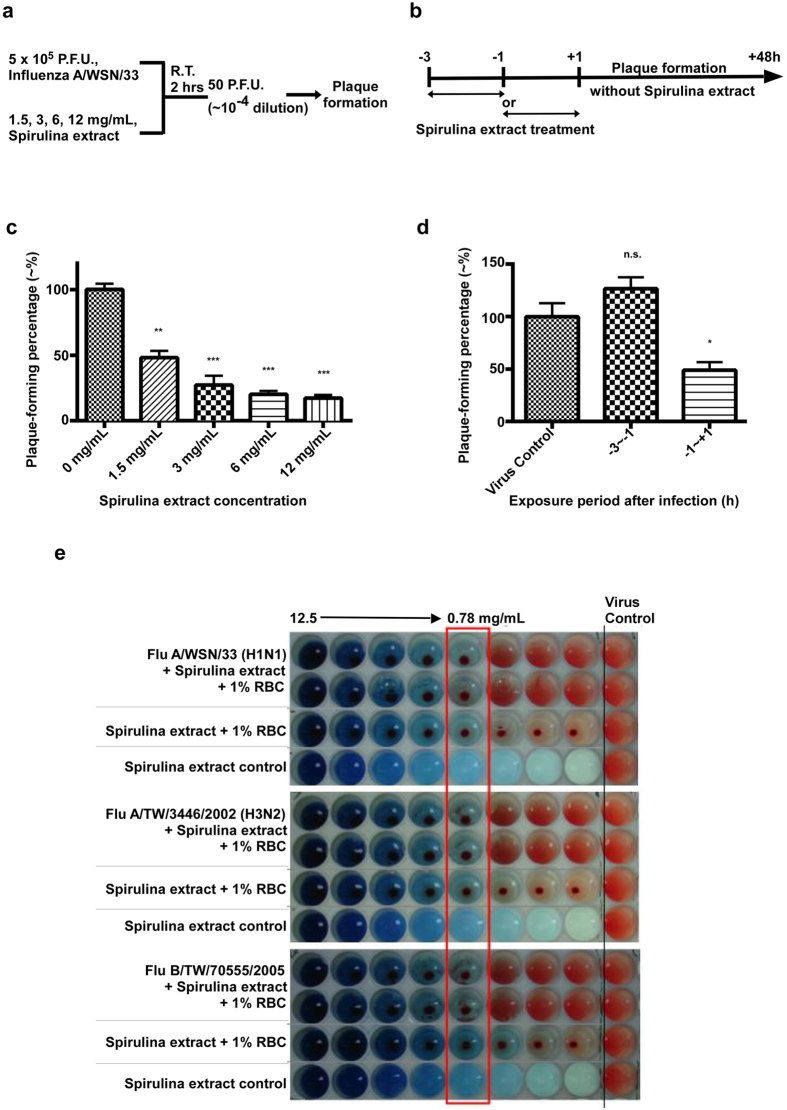
Spirulina extract inhibits influenza virus infection by disrupting hemagglutination. (**a**) 5 × 10^5^ PFUs of the influenza A/WSN/33(H1N1) virus was incubated with 1.5, 3, 6, or 12 mg/mL of Spirulina extract at room temperature for 2 hours, after which cultures underwent a 10^4^-fold dilution to derive samples containing ~50 PFU and negligible amounts of Spirulina extract, which were then used in a plaque assay to assess viral viability. (**b**) Plaque assays showed that plaque formation percentages for Spirulina extract-treated viruses were significantly lower than untreated controls. Statistical analysis was performed with the two-tailed unpaired t-test, and used as the basis to label results as ns, not significant; *P ≤ 0.05; **P < 0.01; ***P < 0.001. (**c**) Monolayer MDCK cells in 6-well plates were either treated with 3 mg/mL of Spirulina extract for 2 hours, after which the culture medium was replaced with fresh medium containing no Spirulina extract (the −3 ~ −1 group), or treated with 3 mg/mL of Spirulina extract just prior to and during viral adsorption (the −1 ~ +1 group). 50 P.F.U. of the influenza A/WSN/33(H1N1) virus were added to cultures at Hour 0, and a plaque assay was conducted to assess viral viability after 48 hours of incubation. (**d**) Plaque assays showed that the plaque formation percentage of the −3 ~ −1 group was comparable to the untreated virus control, while plaque formation was significantly inhibited in the −1 ~ +1 group. Statistical analysis was performed with the two-tailed unpaired t-test, and used as the basis to label results as ns, not significant; *P ≤ 0.05; **P ≤ 0.01; or ***P ≤ 0.001 (**e**) Varying concentrations of Spirulina extract were added to 96-well plates containing 1% of guinea pig RBCs and 4 HA units of influenza A/WSN/33(H1N1), A/TW/3446/02(H3N2), or B/TW/70555/05 viruses. In the virus control column at far right, no Spirulina extract was added. Results showed that concentrations of Spirulina extract above 0.78 mg/mL were capable of inhibiting influenza hemagglutination.

To further elucidate the anti-influenza mechanisms of Spirulina extract during the early stages of virus infection, we conducted experiments to ascertain whether the Spirulina extract acts directly on the influenza virus, or merely primes host cells to prevent viral infection. First, 5 × 10^5^ P.F.U. of the influenza A/WSN/33(H1N1) virus was incubated with 1.5, 3, 6, or 12 mg/mL of Spirulina extract for 2 hours at room temperature. Mixtures were then diluted 10^4^-fold until 50 P.F.U. and negligible concentrations of Spirulina extract remained, and plaque assays were conducted to assess virus viability (Fig. 4a). Compared to untreated controls, viral cultures treated with 1.5, 3, 6, and 12 mg/mL of Spirulina extract respectively lost 51.79%, 72.66%, 79.85%, and 82.73% of plaque formation ability (Fig. 4b). In addition, monolayer MDCK cells in 6-well plates were either pretreated with 3.0 mg/mL of Spirulina extract for 2 hours, after which the culture medium was changed to fresh medium containing no Spirulina extract (the −3 ~ −1 group), or treated with 3 mg/mL of Spirulina extract prior to or at the same time as virus adsorption (the −1 ~ +1 group). After 50 P.F.U. of the influenza A/WSN/33(H1N1) virus strain was added and viral adsorption conducted for two hours at 37 °C, 3 mL of E_0_ medium containing 0.3% of agarose was added to cultures, which were then incubated for 48 hours at 37 °C (Fig. 4c). The plaque formation percentage of the −3 ~ −1 group was 126.5% as compared to the untreated virus control; however, the plaque formation percentage of the −1 ~ +1 group was only 48.9% as compared to the untreated control (Fig. 4d). These results indicate that Spirulina extract likely affects influenza virus directly, rather than priming host cells to prevent viral infection. Considering that the Spirulina extract primarily inhibited the replication of influenza viruses at an early stage, we believed that hemagglutinin could be a potential target for the Spirulina extract, as this surface glycoprotein of the influenza virus is responsible for viral attachment and adsorption during the early phase of infection. Therefore, we conducted a hemagglutination inhibition assay using guinea pig red blood cells (RBCs). The hemagglutinin on influenza viral surfaces can agglutinate RBCs through sialic acid-linked cell surface receptors, while virus particles can also bind to RBCs via hemagglutinin-sialic acid receptor interaction to form distinctive lattices that can be clearly observed in round-bottom microplates. By contrast, RBCs will precipitate to form a spot in the absence of agglutination. We found that concentrations of 0.78 mg/mL or higher of Spirulina extract interrupted viral hemagglutination for the influenza A/WSN/33(H1N1) virus, and similar results were observed for the influenza A/TW/3446/2002(H3N2) and B/TW/70555/2005 viruses as well (Fig. 4e). This indicates that Spirulina extract can inhibit influenza virus strains by affecting hemagglutination at an early stage of virus infection.

### Spirulina extract improves survival in influenza-infected mice

**Figure 5:**
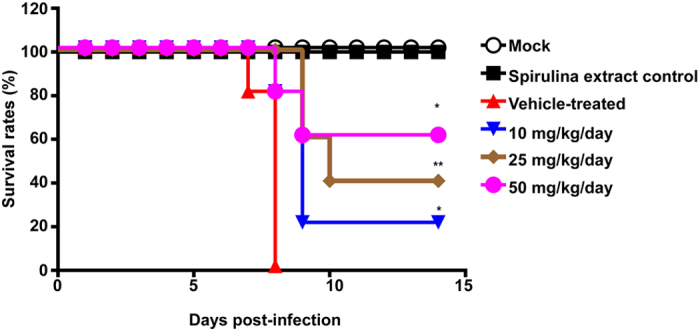
Spirulina extract improves survival rates in influenza-infected mice. Six-week-old female BALB/c mice were inoculated with 2 × 10^4^ PFUs of the influenza A/WSN/33(H1N1) virus, after receiving Spirulina extract 4 hours prior to infection by oral gavage. Spirulina extract was again administered at 6 hours post-infection and twice daily thereafter for 4 days, at respective daily doses of 10, 25, or 50 mg/kg. Survival rates were improved in mice treated with Spirulina extract, as compared to vehicle-treated controls that did not receive Spirulina extract. Statistical analyses of the survival curves were performed using the Log-rank (Mantel-Cox) test compared each Spirulina extract treated group to the vehicle-treated group (n = 5 mice per group). *P < 0.05, **P < 0.01.

Considering that Spirulina extract was well tolerated without any obviously toxicity in animal models, we conducted anti-influenza efficacy tests of Spirulina extract in a mouse model. 6-week-old female BALB/c mice were treated with 5, 12.5, or 25 mg/kg of Spirulina extract by oral gavage at 4 hours prior to infection. Mice were subsequently subjected to intranasal inoculation with the influenza A/WSN/33 (H1N1) virus (2.0 × 10^4^ PFU/mouse), and 6 hours later, a second dose of Spirulina extract was given. Over the next 4 days, mice received two daily doses of Spirulina extract, amounting to a total dose of 10, 25, or 50 mg/kg/day. Survival rates were monitored for 14 days post-infection (Fig. 5), with the day of inoculation defined as Day 0. Results showed that oral administration of Spirulina extract improved survival rates as compared to untreated controls; the survival rates for mice treated with 0, 10, 25, or 50 mg/kg/day of Spirulina extract were respectively 0%, 20%, 40%, and 60% over the 14-day observation period, suggesting that Spirulina extract can substantially improve outcomes in influenza infection.

## Discussion

Many societies around the world live in close proximity to domestic poultry and livestock, which can increase the chances of viral genome reassortment between influenza viruses from different host species. Combined with the convenience of global travel, this means that influenza viruses can evolve and spread more readily than ever before, and the next influenza pandemic is not a question of if, but when^1^,^13^. In the event of an outbreak, vaccine development often lags disease spread, and viral antigenic shift means that newly developed vaccines can become obsolete in a few years^1^. There are also increasing concerns for the efficacy of anti-viral drugs, as resistant influenza strains continue to emerge^6^,^14^; moreover, safety issues have been reported for oseltamivir^15^, one of the most widely used NA inhibitors for influenza prevention and treatment. Novel anti-influenza therapies with a broad range of efficacy are needed, and in this study, we examined the effects of a cold water extract of Spirulina against influenza pathogenicity. Cold water extraction helps to preserve the bioactivity of soluble substances such as proteins and some polysaccharides.

The cold water extract of Spirulina was found to be safe and well-tolerated by Sprague-Dawley rats at doses as high as 5,000 mg/kg for acute toxicity, which surpasses the highest human dose described in the literature thus far (20 g/day of *A. platensis* powder for 6-kg infants)^16^. As the Spirulina cold water extract is about 20% of the biomass for *A. platensis* powder, the aforementioned 20 g/day dose in 6-kg infants corresponds to 3,333 mg/kg/day of *A. platensis* powder, or equal to 666.6 mg/kg/day of Spirulina extract. The conversion factor from human to rat doses is 6.17, meaning that this dose translates to rats is 4,113 mg/kg/day. The median dose of 3,000 mg/kg/day was used to evaluate subacute toxicity in rats over a 14-day period in this study. The doses tested in rats roughly translate to a single dose of 50 g or a daily dose of 30 g for 14 days in a 60-kg human. Considering that the EC_50_ for Spirulina extract ranges from 1.0–1.3 mg/mL *in vitro*, and that doses of 10–50 mg/kg/day were sufficient to improve survival in flu-infected mice which is equalled to 243.3 mg/kg/day in human, it is unlikely that the high doses used in the toxicity studies would ever be applied in a clinical setting. Small scale clinical trials have shown that a daily 50-mL dose of Spirulina hot water extract enhanced interferon-γ production and natural killer (NK) cell functions in healthy male volunteers^17^, while a daily 2,000 mg dose of Spirulina significantly reduced IL-4 levels to modulate cytokine profiles in patients with allergic rhinitis^18^. Furthermore, rhinitis patients treated with a tablet containing just 370 mg of powdered Spirulina exhibited significant improvement in symptoms^19^. This shows that Spirulina extract may exert therapeutic effects at doses far below those tested and found to be safe in our animal toxicity studies.

The anti-viral potential of *A. platensis* has been mentioned previously, and substances such as calcium spirulan (Ca-SP) from *A. platensis* hot water extract were found to be capable of inhibiting the infection and replication of several enveloped viruses *in vitro*, including the influenza A virus, the type 1 human immunodeficiency virus (HIV-1), the type 1 herpes simplex virus (HSV-1), the human cytomegalovirus, the measles virus, and the mumps virus^20^,^21^,^22^,^23^. Braun-type lipoproteins from *A. platensis* ethanol extracts were also found to have immuno-stimulatory effects on monocytes and macrophage cells, and a recent study showed that they may activate innate immunity in mice to protect against the severe pathogenicity caused by influenza virus A (H1N1)^24^. The red fluorescent protein allophycocyanin, purified from *A. platensis*, has previously been shown to inhibit EV71 RNA synthesis and viral plaque formation^25^. And an aqueous extract of *A. platensis* was found to inhibit HIV-1 replication in human T-cells, peripheral blood mononuclear cells, and Langerhans cells^26^. Although the Ca-SP and Braun-type lipoproteins extracted from *A. platensis* demonstrate similar anti-influenza activity as the Spirulina cold water extract described in this study, there are still major differences between these substances. For example, the anti-viral activities of Ca-SP are heat-stable, whereas a hot water extract of the Spirulina extract described in this study yielded no anti-viral activities (Supplementary Table S4), suggesting that the anti-viral properties of the Spirulina cold water extract are not conferred by Ca-SP. The ethanol extract of *A. platensis*, from which Braun-type lipoproteins were extracted, would likely have different constituents from a cold water extract, due to differences in hydrophilicity on extraction. Furthermore, Braun-type lipoproteins were shown to protect mice from influenza infection through immunomodulation, unlike the direct anti-viral activity seen with the Spirulina extract in this study.

For future deployment in clinical trials, chemical characterization and active compound evaluation of the Spirulina extract will be needed, as well as appropriate quality control measures. A complete chemical analysis and elucidation of active compounds would require a significant amount of time and effort, different solvents, fractionation techniques, and analytical equipment would be needed, which is currently beyond the scope of this study. However, we have managed to assemble a basic picture of the chemical components contained in the Spirulina cold water extract, which revealed the contents to be 39.33 ± 5.6% of protein, 11.79 ± 5.7% of polysaccharides, 19.29 ± 2.7% of nucleic acids, 5 ± 1% of water, 1.2 ± 0.3% of ash, and ~23.39% of other or unknown components (Supplementary Table S3). C-phycocyanin makes up about 50% of the protein fraction would be a major component of the Spirulina cold water extract, while allophycocyanin takes up about 10%. In terms of quality control, the identification and quantification of active compounds would be fairly useful to maintain consistency between batches of the Spirulina extract, but this is not feasible as yet. Documentation from Far East Bio-Tec Co., the supplier of the Spirulina cold water extract, states that the fluorescence protein C-phycocyanin is used as a surrogate parameter for quality control purposes as of now. As with active compounds in the Spirulina extract for anti-influenza activity, C-phycocyanin is also susceptible to heat, therefore a quality index of 18–22% C-phycocyanin content in Spirulina extract has been set as a measure of long-term stability. In addition, anti-influenza activity of the Spirulina cold water extract (against influenza A/WSN/33 virus) as evaluated by the neutralization test was used as a bioassay control in our study to monitor efficacy between different batches of Spirulina extract, as well as long-term stability.

We have successfully separated the Spirulina extract into several different fractions, deriving a high molecular weight fraction of components >100 kDa; a polysaccharide-rich fraction from 70% ethanol precipitation; a negatively-charged molecule fraction (comprised of negatively-charged proteins, nucleotides, and polysaccharides) from DEAE ion-exchange column fractionation; a protein-depleted fraction from hot water extraction (HWE), and purified protein fractions containing only C-phycocyanin or allyphycocyanin (Supplementary Table S4). These fractions were tested against the influenza A/WSN/33 virus in anti-influenza neutralization tests, and the results are shown in Supplementary Table S4. The results of the neutralization tests suggest that the active compound(s) responsible for anti-influenza activity are likely to be high molecular weight (>100 kDa), heat-susceptible, and negatively charged polysaccharide(s). We will continue to examine each of these fractions in detail, and hope to elucidate the active substances involved in the near future.

On the other aspect, about 20% of Spirulina extract is made up of the protein C-phycocyanin, which has been reported to downregulate expression of the inflammatory factors iNOS and COX-2 in macrophages or lung tissue^27^,^28^,^29^. C-phycocyanin may also act as a selective COX-2 inhibitor to reduce inflammation^30^. In a salicylate-induced tinnitus mouse model, oral administration of C-phycocyanin was found to downregulate COX-2 mRNA expression in the cochlea and inferior colliculus^31^. It is currently believed that high virus load and a disproportionate host immune response are the two main factors driving influenza pathogenesis^7^,^8^,^32^, and thus the COX-2 inhibitory capabilities and anti-viral properties of the Spirulina extract might potentially act in tandem to improve outcomes in infected patients.

In conclusion, we show here that a cold water extract of Spirulina (*Arthrospira platensis*) was safe and well-tolerated in animal toxicity studies, and significantly inhibited virus infection and replication in a broad range of influenza viruses, including oseltamivir-resistant strains. Survival was also improved in influenza-infected mice treated with Spirulina extract. We found that the Spirulina extract acts by blocking hemagglutination of virus particles to inhibit influenza virus strains. Although it is possible that other anti-influenza mechanisms may be involved, and further research studies will certainly be needed, the fact is that anti-flu therapeutics are urgently needed due to the increasing prevalence of drug-resistant influenza strains. With a long history of food use, high tolerated dose, and broad spectrum of anti-influenza activities, Spirulina extract may serve not only as a viable therapy for the treatment of influenza, but also as a potential prophylaxis for the prevention of disease.

## Methods

### Cold water extraction of Spirulina (*Arthrospira platensis*)

The cold water extract of Spirulina was supplied by FEBICO (Far East Bio-Tec Co., Taipei, Taiwan). The Spirulina extract was prepared as follows: *A. platensis* powder was suspended in pure water with a ratio of 1:10, and the suspension was then rapidly frozen in a refrigerator below −20 °C to form a pellet. The pellet was subsequently thawed at 0–4 °C until completely de-frozen, and insoluble substances were removed by centrifugation for one hour. The supernatant was then lyophilized with freeze driers to derive powdered Spirulina extract containing concentrated levels of soluble biologically active substances.

### Cell cultures and influenza viral strains

Madin-Darby canine kidney (MDCK) cells were cultured in Dulbecco’s Modified Eagle’s Medium (DMEM) (Gibco, Gaithersburg, MD, USA), supplemented with 10% fetal bovine serum (Manufacturer, City, Country). Cells were maintained at 37 °C in a 5% CO_2_ atmosphere. The influenza A/TW/3446/02(H3N2), A/TW/438/07(H3N2), A/TW/70058/09(H1N1), A/TW/147/09(H1N1), A/TW/126/09(H1N1), B/TWN/70555/05, and A/TW/04/2014(H7N9) viruses were obtained from Chang Gung Memorial Hospital, and influenza A/WSN/33(H1N1) virus was purchased from the American Type Culture Collection (ATCC). Influenza virus strains were propagated in MDCK cells, and influenza A/TW/04/2014(H7N9) virus experiments were performed in a Biosafety Level 3 facility, while experiments with other influenza virus strains were performed in a Biosafety Level 2 facility, in accordance with governmental and institutional guidelines.

### Plaque reduction assay

Monolayer MDCK cells in 6-well plates (1 × 10^6^ cells/well) were infected with the respective influenza virus strains, with about 50 plaque-forming units (P.F.U.)/well. Virus was allowed to adsorb to the cells for one hour at room temperature, and serial dilutions (0.375, 0.75, 1.5, or 3.0 mg/mL) of the Spirulina extract were then added respectively to each well at the same time for one hour. The cultures were subsequently overlaid with 3 mL of E0 DMEM containing 0.3% agarose and each dilution of Spirulina extract, then incubated for 48 hours at 37 °C. Afterwards, cells were fixed with 10% formaldehyde for 1 hour, and then stained with 0.5% of crystal violet. Plaques were visualized and counted to calculate half maximal effective concentration (EC_50_) values.

### Cytotoxicity assay of Spirulina extract

MDCK cells were seeded in 96-well microplates (30,000 cells/well), and cultured in a humidified incubator for 24 hours. Culture media were then replaced with media containing Spirulina extract, at concentrations ranging from 0.048 to 50 mg/mL. A control group cultured in DMEM without Spirulina extract, and a blank group containing neither cells nor culture media, were also included. Cells were incubated for 72 hours, after which the culture medium was discarded and 20 μL of 3-(4,5-dimethylthiazol-2-yl)-2,5-diphenyltetrazolium bromide (MTT) solution (1 mg/mL in DMEM) was added to each well. Cells were incubated for an additional 4 hours, and the MTT solution was then removed; subsequently, 200 μL of 0.04 N HCl in isopropanol was added to each well to dissolve the formazan crystals. Plates were then read on a microplate reader (BIOtAK, Bristol, UK) at absorbance of 570 nm (OD_570_). Four wells were used for each concentration of Spirulina extract, and the concentration resulting in 50% cell death compared with untreated controls (CC_50_) was calculated according to the Reed-Muench method.

### Influenza virus neutralization test

The respective influenza virus strains were added to confluent MDCK cells in 96-well plates, which were then treated with serial concentrations of Spirulina extract and subsequently overlaid with 200 μL/well of E_0_ DMEM. After incubation at 37 °C for 64 hours, cells were fixed with 100 μL of 0.5% formaldehyde for one hour at room temperature, and then stained with 0.1% of crystal violet for 15 minutes at room temperature. Plates were then washed and dried, and the cellular densities of each well were measured with a microplate reader (BIOtAK) at OD_570_. The concentration of Spirulina extract required to reduce virus-induced cytopathic effects (CPE) by 50% compared to untreated controls was expressed as the EC_50_.

### Virus yields percentage with TCID_50_ evaluation

Influenza A/TWN/04/2014(H7N9) virus was co-incubated with 0, 0.2, 1, and 2 mg/mL of Spirulina extract for 60 mins. The mixtures were then serially-diluted 10-fold in E_0_-DMEM, and 100 μl of each diluted mixture was added to the corresponding wells of 96-well plates containing a monolayer of MDCK cells and 100 μl of E_0_ DMEM. Cultures were incubated for 3 days and then monitored with a microscope to calculate the TCID_50_ of each group according to the Reed-Muench method. Results from the 0.2, 1, and 2 mg/mL Spirulina extract-treated groups were compared to the 0 mg/mL virus control group to evaluate the virus yield percentage.

### Time of addition assay

MDCK cells in 6-well plates were infected with influenza A/WSN/33(H1N1) virus at a multiplicity of infection (MOI) of 2, and then treated with Spirulina extract (2.5 mg/mL) at different timepoints post-infection. At 9 hours post-infection, the culture medium was replaced with E_0_ overlay medium (DMEM containing 100 U/mL of penicillin, 100 μg/mL of streptomycin, 2 mM of L-glutamine, and 0.1 mM of non-essential amino acid mixture), and supernatants from each well were collected at 12 hours post-infection. Virus titers were determined by plaque assay as previously described^11^.

### Hemagglutination inhibition assay

Serially diluted influenza viral strains were respectively mixed with an equal volume of 1% guinea pig RBCs suspended in isotonic PBS in round-bottom 96-well plates, which were then incubated for 1 hour at 4 °C. The lowest concentration of virus to cause RBC agglutination was defined as one HA unit, and we subsequently treated 4 HA units of each respective influenza viral strain with serial dilutions of Spirulina extract for one hour at 4 °C, after which the viruses were respectively mixed with an equal volume of 1% guinea pig RBCs suspended in PBS in round-bottom 96-well plates, and incubated for another hour at 4 °C. The HI (hemagglutination inhibition) unit was defined as the lowest concentration of Spirulina extract capable of inhibiting viral hemagglutination.

### Spirulina extract toxicity assays

Spirulina extract was assessed by the Ames test, with 50, 150, 500, 1,500, or 5,000 μg/plate of Spirulina extract added to induce reverse mutations in *Salmonella typhimurium* tester strains, in the absence or presence of S9 metabolic activation, in order to assess genotoxicity. A chromosome aberration assay was conducted in CHO cells treated with 50, 150, 500, 1,500, or 5,000 μg/ml of Spirulina extract in the absence or presence of S9, to evaluate the clastogenic potential of Spirulina extract. Acute toxicity studies of Spirulina extract were also conducted *in vivo*. A 14-day acute oral toxicity study was conducted in Sprague-Dawley rats, using doses of 0, 1,000, 3,000, or 5,000 mg/kg of Spirulina extract. Clinical signs, mortality, and body weight were monitored for 15 days, and necropsies were conducted at the end of the study. We further conducted a 14-day repeated dose (subacute) oral toxicity study in Sprague-Dawley rats, which were consecutively given 750, 1,500, or 3,000 mg/kg/day of Spirulina extract for 14 days. Body weight and mortality were tracked, and ophthalmologic examinations, urinalysis, haematology assays, blood coagulation assays, and serum chemistry assays were also conducted. The toxicology-associated studies were performed by the Center of Toxicology and Preclinical Sciences, Development Center for Biotechnology. All animal experiments were approved by the Institutional Animal Care and Use Committee of the Development Center for Biotechnology, and experimental animals were treated humanely in accordance with the National Institutes of Health guidelines on the ethical use of animals.

### Survival analysis in a mouse model

BALB/c mice were purchased from BioLasco Biotechnology Company (Taipei, Taiwan) and housed in an environmentally-controlled room at a temperature of 25 ± 1 °C with a 12-hour light-dark cycle. Six-week-old female BALB/c mice were respectively treated with 5, 12.5, or 25 mg/kg of Spirulina extract by oral gavage at 4 hours prior to infection, and were subsequently subjected to intranasal inoculation with the influenza A/WSN/33 (H1N1) virus (2.0 × 10^4^ PFU/mouse). Mice received a second dose of Spirulina extract at 6 hours post-infection, and the same doses were administered twice daily over the next 4 days, amounting to a daily dose of 10, 25, or 50 mg/kg/day of Spirulina extract. The day of inoculation was defined as Day 0, and survival rates were monitored for up to 14 days post-inoculation. Animal experimental protocols were conducted in accordance with the policies and procedures described in the Guide for the Care and Use of Laboratory Animals of the National Institutes of Health, and were approved by the Chang Gung University review committee (IACUC approval number CGU11-109).

### Statistical analysis

Data are expressed as mean ± SD. Statistical analysis was performed using GraphPad Prism software. Statistical significance was labelled as ^*^P ≤ 0.05, ^**^P ≤ 0.01, and ^***^P ≤ 0.001.

## Additional Information

**How to cite this article**: Chen, Y.-H. *et al.* Well-tolerated Spirulina extract inhibits influenza virus replication and reduces virus-induced mortality. *Sci. Rep.* **6**, 24253; doi: 10.1038/srep24253 (2016).

## Supplementary Material

Supplementary Information

## Figures and Tables

**Table 1:** Antiviral activity of Spirulina extract against influenza virus strains *in vitro*.

	EC_50_ (mg/mL)	CC_50_ (mg/mL)	SI
A/WSN/33(H1N1)	1.17 ± 0.09	23.80	20.34
A/TW/3446/2002(H3N2)	0.98 ± 0.24	23.80	24.28
B/TW/70555/2005	1.33 ± 0.15	23.80	17.89
A/TW/70058/2009(H1N1)*	1.17 ± 0.08	23.80	20.34
A/TW/147/2009(H1N1)*	0.98 ± 0.10	23.80	24.28
A/TW/126/2009(H1N1)	0.58 ± 0.02	23.80	41.03

EC_50_: 50% effective concentration, based on the inhibition of viral plaque formation.

CC_50_: 50% cytotoxic concentration, based on the MTT assay.

SI: selectivity index, a ratio of CC_50_ to EC_50_.

^*^Oseltamivir-resistant influenza strains.

All data represent mean ± SD for three independent experiments.
