# Supplementary material for: Well-tolerated Spirulina extract inhibits influenza virus replication and reduces virus-induced mortality
Source: Sci Rep. 2016 Apr 12;6:24253. doi: 10.1038/srep24253 (PMC4828654; doi:10.1038/srep24253)
Supplement: Supplementary Information [file srep24253-s1.pdf]

Well-tolerated Spirulina extract inhibits influenza virus replication and reduces virus-induced mortality

Yi-Hsiang Chen[1,3], Gi-Kung Chang[1], Shu-Ming Kuo[1], Sheng-Yu Huang[1,3], I-Chen Hu[5], Yu-Lun Lo[1], and Shin-Ru Shih[1,2,3,4,*]

[1]Research Centre for Emerging Viral Infections,

[2]Department of Medical Biotechnology and Laboratory Science, and

[3]Graduate Institute of Biomedical Sciences, College of Medicine, Chang Gung University, Taoyuan, Taiwan, ROC.

[4]Clinical Virology Lab, Department of Clinical Pathology, Chang Gung Memorial Hospital, Taoyuan, Taiwan, ROC.

[5]Institute of Bioinformatics and Structural Biology, National Tsing-Hua University, Hsinchu, Taiwan, ROC.

[*]Correspondence should be addressed to: S.R.S (email: srshih@mail.cgu.edu.tw)

Supplementary Information

Supplementary Table S1. Hematology results in rats (Mean ± SD, N=10)

Parameters	Sex	Dose (mg/kg/day)			
		0	750	1500	3000
WBC (10^3/μL)	Male	11.058 ± 3.260	11.769 ± 3.089	14.721 ± 3.362	13.434 ± 4.067
	Female	11.158 ± 3.107	11.104 ± 3.626	11.387 ± 2.467	11.280 ± 4.141
RBC (10^6/μL)	Male	7.867 ± 0.238	8.066 ± 0.456	7.889 ± 0.375	7.809 ± 0.236
	Female	8.447 ± 0.488	8.287 ± 0.585	8.558 ± 0.555	8.392 ± 0.517
Hb (g/dL)	Male	14.83 ± 0.52	15.22 ± 0.94	15.19 ± 0.57	15.00 ± 0.57
	Female	15.64 ± 0.91	15.47 ± 1.19	15.98 ± 0.78	15.69 ± 0.83
Ht (%)	Male	46.98 ± 1.41	48.19 ± 2.82	47.80 ± 1.75	47.14 ± 1.83
	Female	48.35 ± 2.94	47.64 ± 3.34	48.93 ± 2.34	48.23 ± 2.51
MCV (fl)	Male	59.66 ± 0.88	59.76 ± 1.13	60.58 ± 1.01	60.37 ± 1.40
	Female	57.24 ± 1.60	57.52 ± 0.88	57.24 ± 1.75	57.52 ± 1.63
MCH (pg)	Male	18.86 ± 0.40	18.87 ± 0.41	19.26 ± 0.27	19.22 ± 0.47
	Female	18.49 ± 0.50	18.68 ± 0.33	18.67 ± 0.68	18.71 ± 0.46
MCHC (%)	Male	31.59 ± 0.46	31.58 ± 0.27	31.81 ± 0.41	31.81 ± 0.38
	Female	32.32 ± 0.37	32.45 ± 0.46	32.63 ± 0.64	32.53 ± 0.46
Platelet (10^3/μL)	Male	1134.0 ± 155.0	1152.4 ± 156.4	1088.8 ± 168.8	1150.2 ± 147.8
	Female	1219.0 ± 86.0	1197.0 ± 164.8	1244.3 ± 104.5	1209.2 ± 106.5

*: $P < 0.05$

Supplementary Table S2. Serum Chemistry in rats (Mean ± SD, N=10)

Parameters	Sex	Dose (mg/kg/day)			
		0	750	1500	3000
AST (U/L)	Male	128.2 ± 40.8	101.3 ± 10.3*	104.7 ± 18.3	102.1 ± 12.9*
	Female	105.0 ± 51.5	87.5 ± 8.2*	90.0 ± 21.1	94.2 ± 16.5
ALT (U/L)	Male	34.0 ± 4.1	31.5 ± 5.1	30.2 ± 4.2	27.9 ± 4.7
	Female	34.8 ± 38.7	23.8 ± 3.1	23.8 ± 5.7	26.5 ± 5.9
ALP (U/L)	Male	193.6 ± 25.4	169.0 ± 17.5	169.6 ± 26.8	164.6 ± 38.1
	Female	100.1 ± 21.8	94.1 ± 22.3	98.0 ± 28.1	94.1 ± 19.9
Total proteins (g/dL)	Male	6.59 ± 0.21	6.52 ± 0.36	6.47 ± 0.31	6.56 ± 0.35
	Female	7.14 ± 0.54	7.28 ± 0.30	7.37 ± 0.32	7.16 ± 0.24
Uric acid (mg/dL)	Male	2.67 ± 0.59	2.71 ± 0.53	2.68 ± 0.56	2.89 ± 1.02
	Female	2.44 ± 0.62	2.87 ± 0.50	2.35 ± 0.34	2.36 ± 0.45
γGT	Male	0.867 ± 0.454	0.767 ± 0.565	0.713 ± 0.635	0.506 ± 0.434
	Female	0.651 ± 0.644	1.083 ± 0.749	0.681 ± 0.500	0.785 ± 0.608
Glucose (mg/dL)	Male	146.4 ± 26.9	149.2 ± 24.8	155.5 ± 24.7	165.6 ± 43.4
	Female	143.4 ± 37.6	162.5 ± 38.3.	142.2 ± 34.6	144.8 ± 22.5
Creatinine (mg/dL)	Male	0.41 ± 0.06	0.41 ± 0.03	0.42 ± 0.04	0.43 ± 0.05
	Female	0.50 ± 0.05	0.47 ± 0.05	0.49 ± 0.07	0.47 ± 0.07

*: P<0.05

Supplementary Table S3. Composition analysis of Spirulina extract

	Content~% (w/w)
Protein	39.33 ± 5.6
Polysaccharide	11.79 ± 5.7
Nucleic acid	19.29 ± 2.7
Water	5 ± 1
Ash	1.2 ± 0.3
Others	23.39

Protein content determined by Bradford assay

Polysaccharide content determined by the phenol-sulfric acid method

Nucleotide content determined by OD260 absorbance

Water content determined by the loss on drying method

Ash content determined by incineration at 600°C for 7 hrs.

Supplementary Table S4. Antiviral activity of the further fractionation of Spirulina extract against Flu A/WSN/33 virus

	**EC50 (mg/mL)**
Spirulina extract	0.955 ± 0.052
Spirulina extract (> 100KD)	0.657 ± 0.202
Spirulina extract (< 100KD)	Not detected
Spirulina extract (Ethanol prec.)	0.602 ± 0.04
Spirulina extract (DEAE)	0.668 ± 0.033
Spirulina extract (TCA)	0.835 ± 0.02
Spirulina extract (HWE)	Not detected
C-phycocyanin	Not detected
Allyphycocyanin	Not detected

>100KD and <100KD: Fractionized with molecular size at 100KD by “Amicon Ultra-15 Centrifugal Filter Unit with Ultracel-100 membrane;
Ethanol prec.: Fractionized with 70% ethanol precipitation;
DEAE: Fractionized with DEAE gel (SepharoseTM Fast Flow) for negative charge molecules;
TCA: 10%Trichloroacetic acid precipitation to remove protein fraction;
HWE: Hot water extraction with 99°C for 1hr. to denature proteins fraction;
EC50: 50% effective concentration, based on neutralization test;
All data represent mean ± SD for three independent experiments.
